# Regulatory Role of RNA N^6^-Methyladenosine Modification in Bone Biology and Osteoporosis

**DOI:** 10.3389/fendo.2019.00911

**Published:** 2020-01-10

**Authors:** Xuejiao Chen, Wenfeng Hua, Xin Huang, Yuming Chen, Junguo Zhang, Guowei Li

**Affiliations:** ^1^Center for Clinical Epidemiology and Methodology (CCEM), Guangdong Second Provincial General Hospital, Guangzhou, China; ^2^Department of Laboratory Medicine and Central Laboratories, Guangdong Second Provincial General Hospital, Guangzhou, China; ^3^Department of Medical Statistics and Epidemiology, School of Public Health, Sun Yat-sen University, Guangzhou, China; ^4^Department of Health Research Methods, Evidence, and Impact (HEI), McMaster University, Hamilton, ON, Canada

**Keywords:** RNA N^6^-methyladenosine modification, m^6^A writers, m^6^A erasers, bone development, osteoporosis

## Abstract

Osteoporosis is a metabolic skeletal disorder in which bone mass is depleted and bone structure is destroyed to the degree that bone becomes fragile and prone to fractures. Emerging evidence suggests that N^6^-methyladenosine (m^6^A) modification, a novel epitranscriptomic marker, has a significant role in bone development and metabolism. M^6^A modification not only participates in bone development, but also plays important roles as writers and erasers in the osteoporosis. M^6^A methyltransferase METTL3 and demethyltransferase FTO involves in the delicate process between adipogenesis differentiation and osteogenic differentiation, which is important for the pathological development of osteoporosis. Conditional knockdown of the METTL3 in bone marrow stem cells (BMSCs) could suppress PI3K-Akt signaling, limit the expression of bone formation-related genes (such as Runx2 and Osterix), restrain the expression of vascular endothelial growth factor (VEGF) and down-regulate the decreased translation efficiency of parathyroid hormone receptor-1 mRNA. Meanwhile, knockdown of the METTL3 significantly promoted the adipogenesis process and janus kinase 1 (JAK1) protein expression via an m^6^A-dependent way. Specifically, there was a negative correlation between METTL3 expression and porcine BMSCs adipogenesis. The evidence above suggested that the relationship between METTL3 expression and adipogenesis was inverse, and osteogenesis was positive, respectively. Similarly, FTO regulated for BMSCs fate determination during osteoporosis through the GDF11-FTO-PPARγ axis, prompting the shift of MSC lineage commitment to adipocyte and inhibiting bone formation during osteoporosis. In this systematic review, we summarize the most up-to-date evidence of m^6^A RNA modification in osteoporosis and highlight the potential role of m^6^A in prevention, treatment, and management of osteoporosis.

## Introduction

Osteoporosis is a systemic skeletal disease characterized by decrease in bone mineral density (BMD) and deterioration in bone microarchitecture (1, 2). It is a complex multifactorial disorder due to an interaction between genetic and environmental factors, dietary habits, and lifestyle. Patients suffer from chronic pain and decreased quality of life (3). Osteoporotic fractures increase disability, mortality, and health-care cost, especially among elder peoples (4). For example, the cumulative mortality after 1 year of an osteoporotic hip fracture occurrence varies between 20 and 40% (5). Due to its silent nature, osteoporosis is often under-diagnosed and under-managed, which needs immediate attention.

Epigenetics is the study of heritable changes in gene expression that do not involve alterations in the DNA/RNA sequence, including DNA methylation, histone modification, and RNA modification (6, 7). As a consequence of gene–environment interactions, various environmental factors could trigger different epigenetic processes which regulate gene transcription (8, 9). Among them, DNA methylation and demethylation are the most extensively studied, especially alteration in the methylation of cytosine nucleotides in CpG islands located in the promoter region of genes. Hypomethylation of the cytosine bases of the DNA promoter sequence in CpG islands activates gene expression, and hypermethylation silences gene expression (8, 10). Aberrant DNA methylation patterns can result in developmental disorders (11). Modification of histone molecules within chromatin plays important roles in regulating gene expression. Enzymes, including histone acetyltransferases (HAT), histone methyltransferases (HMT), histone deacetylases (HDAC), histone demethylases (HDM), and others, could modify histones to alter gene expression by regulating promoter activity, chromatin structure, dosage compensation, and epigenetic memory, without changes in the nucleic acid sequences (8, 12). Moreover, epigenetic factors are also involved in bone biology and osteoporosis, which play a bridging role between individual genetic aspects and environmental influences (13).

**Figure 1:**
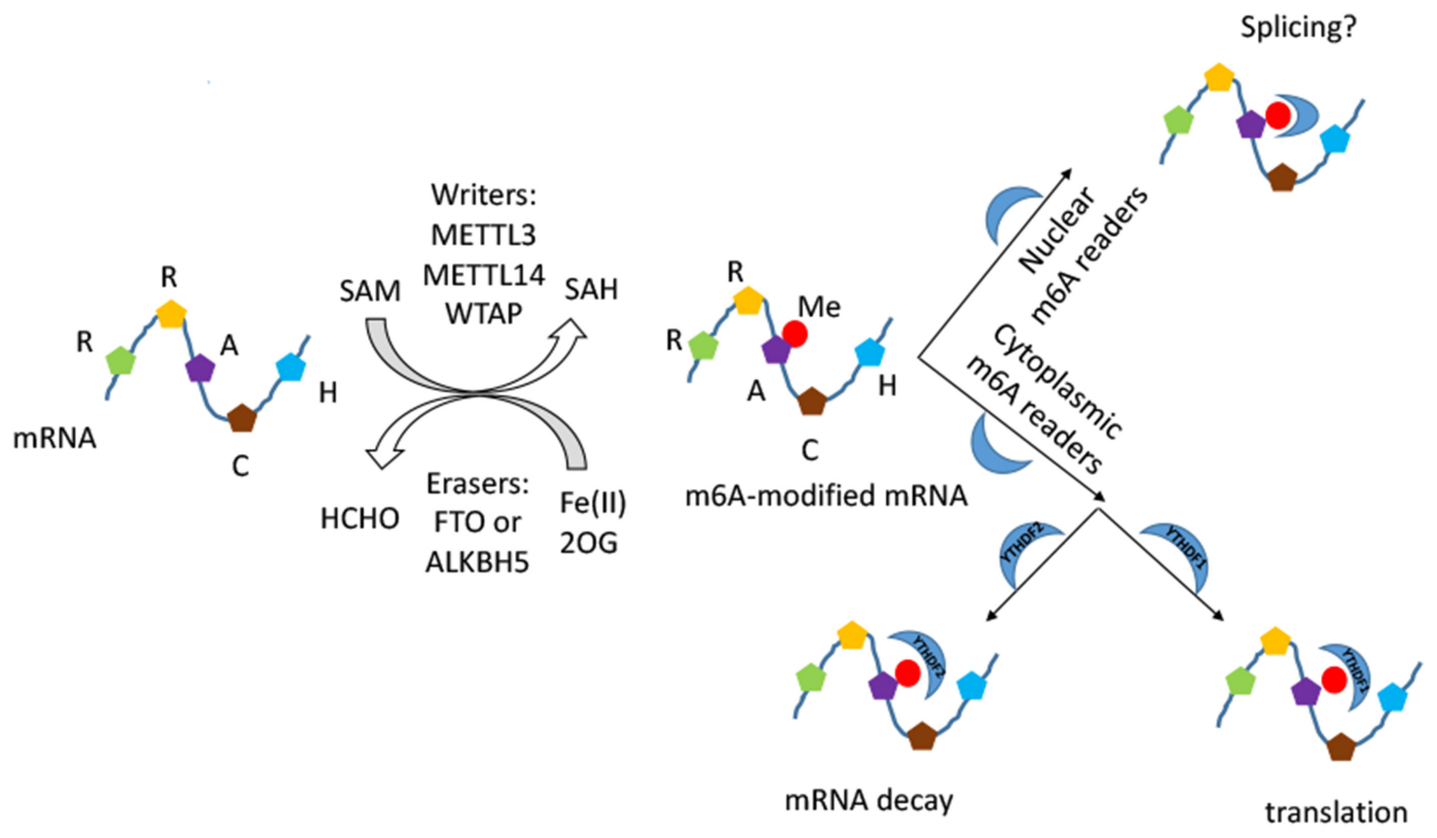
The schematic diagram of m^6^A RNA modification. The process is composed of installation, removal, and identification performed by writers, erasers, and readers. The METTL3–METTL14–WTAP methyltransferase complex catalyses a methyl group transfered from SAM into the N6 position, and the demethylases FTO and ALKBH5 catalyse the oxidative demethylation from methylated adenosine. The methyl group introduced is marked in red. The readers of the YTH domain family are effectors that decode the m6A methylation code and transform it into a functional signal in nucleus and cytoplasm.

RNA modification is another important post-transcriptional regulation, among which N^6^-methyladenosine (m^6^A) modification of mRNA is one of the most highly abundant (14, 15). First reported in 1970s, m^6^A modification was found to have a broad functional influence on stabilizing homeostasis closely correlated to post-transcriptional gene expression regulation, growth and development (14, 16–20). It regulates the metabolic processes of most RNAs, including the pre-mRNA splicing, mRNA export, turnover, and translation of mRNA (18, 21–23). M^6^A modification is tightly closely correlated to fundamental biological processes such as adipogenesis (24–26), mammalian spermatogenesis development (27), RNA dynamics of T cells (28), pluripotency differentiation (29–35), and response to heat shock (36, 37). Moreover, it was found to get involved in the etiology of various diseases including cancers (36, 38, 39), systemic lupus erythematosus (40), rheumatoid arthritis (16), and coronary artery disease (41). It was revealed that m^6^A modification commonly occurred at the consensus motif RRACH (R = A, G; H = A, C, U) (14, 40). The process is catalyzed by the orchestrated action of highly conserved methyltransferase (m^6^A writers) and demethylase (m^6^A erasers) enzymes (42). M^6^A writer is composed of a METTL3 (methyltransferase-like3)-METTL14 (methyltransferase-like 14)-WTAP (Wilm's tumor–associated protein) complex (43–45). Two members of the Fe(II)- and 2-oxoglutarate-dependent oxygenase superfamily, FTO and ALKBH5, act as m^6^A erasers (46). N6-methyladenosine (m^6^A) reader proteins of the YTH family serve as recognition elements for the effector proteins. YTHDF1/3 enhance translation efficiency of methylated mRNAs, while YTHDF2 promotes mRNA decay (6) (Figure 1). Recently, it is demonstrated that the DNA demethylase ALKBH1 play an unexpected role in modulating hypoxia-induced genes in human glioblastoma. M^6^A modification of DNA modification is markedly upregulated and highly associated with the H3K9me3 heterochromatin histone modification in human glioblastoma (47).

It has been shown that DNA methylation and posttranslational histone modification participant in gene expression of bone cells (48, 49). These epigenetic programs are essential for physiological and pathological process, such as bone remodeling and bone metabolic disorders (6, 50). Besides, there is growing evidence that m^6^A modification is a potential pathogenesis mechanism in osteoporosis (51–53). We systematically searched PubMed and EMBASE (up to July 2019) using keywords “(mRNA modifications OR epitranscriptomics OR N6-methyladenosine modification OR m^6^A modification OR m^6^A OR FTO OR Mettl3) AND (bone OR osteoporosis OR bone marrow stem cells OR BMSCs OR bone mineral density OR BMD)” to decipher the role of m^6^A modification in osteoporosis, which might help further understand the pathogenesis of osteoporosis and provide theoretical basis for potential epigenetic-based therapeutics of osteoporosis. The inclusion criteria was: (1) to evaluate the association between RNA N6-methyladenosine modification in bone biology and osteoporosis; (2) full-text articles; (3) sufficient data on the regulatory mechanism. We identified 223 and 103 citations in PubMed and EMBASE, respectively. After removing 32 duplicates, 294 citations remained for title and abstract screening, from which nine articles were retrieved for full text screening (Table 1). Studies excluded due to not associated with RNA N6-methyladenosine modification in bone biology and osteoporosis (*n* = 247), reviews (*n* = 26), meta-analysis (*n* = 2), letters (*n* = 1), case reports (*n* = 2), meeting abstracts (*n* = 4), protocol (*n* = 1), and clinical trials (*n* = 2). Of the nine relevant studies, six were experimental studies, two were the candidate gene association studies and one was genome-wide association study. Based on the nine included studies, we discussed five parts below in detail related to m^6^A modification and osteoporosis in this systematic review.

**Table 1:** Summary of included studies about the regulatory role of m^6^A mRNA modification in osteoporosis.

**References**	**Country**	**Key findings**	**Study type**	**Summarized role of m^**6**^A in osteoporosis**
Tian et al. (54)	China	METTL3 regulates osteogenic differentiation and alternative splicing of Vegfa in bone marrow mesenchymal stem cells.	Experimental study	Bone development; Differentiation of adipocyte and osteoblast.
Yao et al. (55)	China	METTL3 inhibits BMSC adipogenic differentiation by targeting the JAK1/STAT5/C/EBPbeta pathway via an m^6^A-YTHDF2–dependent manner.	Experimental study	Bone development; Differentiation of adipocyte and osteoblast.
Wu et al. (53)	China	METTL3-mediated m(6)A RNA methylation regulates the fate of bone marrow mesenchymal stem cells and osteoporosis.	Experimental study	Bone development; m^6^A writer in osteoporosis; Differentiation of adipocyte and osteoblast.
Tran et al. (56)	Australia	Association between fat-mass-and-obesity-associated (FTO) gene and hip fracture susceptibility.	The candidate gene association study	m^6^A-associated SNPs for bone mineral density or osteoporosis.
Guo et al. (57)	China	The fat mass and obesity associated gene, FTO, is also associated with osteoporosis phenotypes.	The candidate gene association study	m^6^A eraser in osteoporosis; m^6^A-associated SNPs for bone mineral density or osteoporosis; Differentiation of adipocyte and osteoblast.
Shen et al. (51)	China	The GDF11-FTO-PPARgamma axis controls the shift of osteoporotic MSC fate to adipocyte and inhibits bone formation during osteoporosis.	Experimental study	Bone development; m^6^A eraser in osteoporosis.
Mo et al. (52)	China	Genome-wide identification of m(6)A-associated SNPs as potential functional variants for bone mineral density.	Genome-wide association study	m^6^A-associated SNPs for bone mineral density or osteoporosis.
Sachse et al. (58)	UK	FTO demethylase activity is essential for normal bone growth and bone mineralization in mice.	Experimental study	m^6^A eraser in osteoporosis.
McMurray et al. (59)	UK	Pharmacological inhibition of FTO.	Experimental study	m^6^A eraser in osteoporosis.

## M^6^A Modification Regulates Bone Development

M^6^A modification of mRNAs has been discovered as a reversible RNA methylation and is widely conserved in mammalian cells (15, 42, 60). It is the most prevalent and internal modification that is tightly related to fundamental biological processes (Figure 1). M^6^A has recently been reported to play a part in pluripotency differentiation and development of the cell lineage (29, 30, 32, 33), including osteogenic differentiation of bone marrow stem cells (BMSCs) (51, 53, 54). The human skeleton is a metabolically active tissue that undergoes continuous turnover and remodeling throughout life (48). Under homeostatic conditions, there is a delicate balance between osteoblast-mediated bone regeneration and osteoclast-mediated bone resorption (61, 62). Abnormalities of this process can produce a variety of skeletal disorders (63). BMSCs, also known as bone marrow-derived mesenchymal stem cells, are multipotent stromal cells with the ability of differentiating into osteoblast, chondrocyte, and adipocyte both *in vitro* and *in vivo* (64). In normal conditions, that would be a dynamic equilibrium for their differentiation of adipocytes and osteoblasts (65).

**Figure 2:**
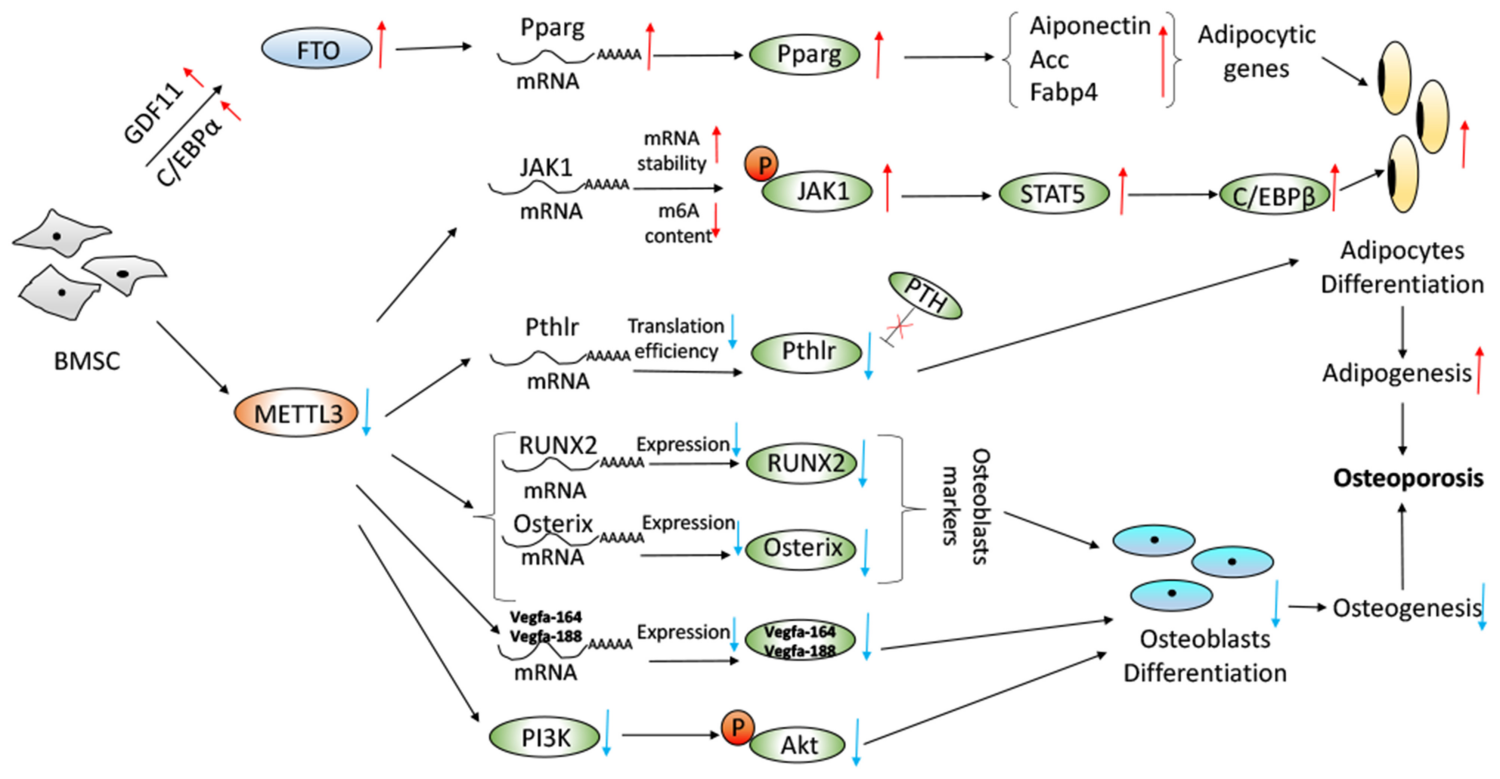
Proposed model depicting regulation and role of METTL3 and FTO in BMSC adipogenesis and osteogenesis with regard to osteoporosis. Working model of the mechanism of METTL3 knockdown increased JAK1/STAT5/C/EBPb pathway, decreased PTH/Pth1r signaling axis, decreased expression of RUNX2, Osterix, and Vegfa and decreased PI3K-Akt signaling pathway. Overexpression of FTO in a GDF11-C/EBPα-dependent mechanism resulted in increase of the Pparg mRNA and the promotion of adipogenesis.

Recently, Yao et al. found that METTL3 plays an important role in BMSCs differentiation and adipogenesis. There was a negative correlation between METTL3 expression and porcine BMSCs (pBMSCs) adipogenesis (55). Specifically, METTL3 inhibited pBMSCs adipogenic differentiation by targeting the JAK1/STAT5/C/EBPβ pathway *via* an m^6^A-YTHDF2–dependent manner. It was demonstrated that the deletion of METTL3 significantly promoted the pBMSCs adipogenesis process and janus kinase 1 (JAK1) protein expression *via* an m^6^A-dependent way (55) (Figure 2). Similarly, in Tian's study, it was shown that METTL3 was highly expressed in osteogenically differentiated BMSCs (54). METTL3 knockdown limited the expression of vascular endothelial growth factor (VEGF) and its bone formation-related splice variants (Vegfa-164 and Vegfa188) in osteoblast-induced BMSCs, which was implicated in the maturation of osteoblasts, ossification and bone turnover. METTL3 knockdown decreased the expression of bone formation-related genes (such as Runx2 and Osterix), Akt phosphorylation, the alkaline phosphatase activity and the formation of mineralized nodules. PI3K-Akt signaling was suppressed by METTL3 knockdown in BMSCs during the osteogenic differentiation process (54) (Figure 2). Furthermore, METTL3 had a functional role in osteoarthritis progression by regulating NF-kB signaling and extracellular matrix synthesis in chondrocytes (66). RNA demethylase AlkB Homolog 5 (ALKBH5) was amplified in sarcomas and its expression was highly elevated in osteosarcoma patients. Silencing of ALKBH5 inhibited the osteosarcoma growth and migration without affecting the viability of normal human fetal osteoblast cells by sensitizing osteosarcoma cells to DNA damaging agents (67). Moreover, METTL3 promoted osteosarcoma cell progression by regulating the m^6^A level of lymphoid enhancer-binding factor 1and activating Wnt/b-catenin signaling pathway (68).

## M^6^A Writers in Osteoporosis

Wu et al. reported that METTL3-mediated m^6^A RNA methylation could regulate the fate of bone marrow mesenchymal stem cells and osteoporosis (53). Conditional knockout of the m^6^A methyltransferase METTL3 in BMSCs induced pathological features of osteoporosis in mice and resulted in impaired bone formation, incompetent osteogenic differentiation potential, and increased marrow adiposity. Conversely, METTL3 overexpression in BMSCs protected the mice from estrogen deficiency-induced osteoporosis. METTL3 depletion resulted in the decreased translation efficiency of parathyroid hormone receptor-1 mRNA. M^6^A affected both osteogenic and adipogenic differentiation of MSCs through PTH (parathyroid hormone)/Pth1r (parathyroid hormone receptor-1) signaling axis. METTL3 knockout reduced the translation efficiency of MSCs lineage allocator Pth1r, leading to reduction of the global methylation level of m^6^A and disruption of the PTH-induced osteogenic and adipogenic responses. PTH, bone-promoting molecule, stimulates bone formation by activating the HSP90-dependent PERK-EIF2α-ATF4 signaling pathway (69) and increasing the receptor activator of nuclear factor κ-B ligand (RANKL)/osteoprotegerin (OPG) ratio through its receptor (70). M^6^A modification was required for Pth1r translation. Loss of METTL3 changed Pth1r mRNA from the polysome fractions to the sub-polysome fractions (71), leading to slowing down the protein synthesis of Pth1r and blocking the downstream signaling pathways of Pth1r responsive to PTH treatment (53). The regulatory mechanism was shown in Figure 2.

## M^6^A Erasers in Osteoporosis

As an m^6^A eraser, FTO was associated with osteoporosis phenotypes (57). It was founded that the whole body FTO knockout mice appeared as immediate postnatal growth retardation with shorter body length, lower body weight, and lower bone mineral density (BMD) (72). As Sachse et al. showed that FTO catalytic was essential for normal bone growth and mineralization but was not required for normal body composition except for normal body size and viability (58). They found both BMD and BMC (bone mineral content) were reduced in FTO knockout mice, which was comparable to that seen in osteoporosis. It was indicated that a relatively small amount of catalytic activity, roughly 20–50%, was sufficient to rescue the bone phenotype (58).

Moreover, Shen et al. have reported that FTO was a regulator for BMSCs fate determination during osteoporosis, with a rise in bone marrow in a growth-differentiation factor 11 (GDF11)-C/EBPα-dependent mechanism. Increased serum GDF11 concentration was associated with a high prevalence of osteoporosis by stimulating osteoclastogenesis and inhibiting osteoblast through inducing Smad2/3 phosphorylation (73–75). Peroxisome proliferator-activated receptor gamma (PPARγ) promoted the adipocyte differentiation and inhibited osteoblast differentiation from BMSCs (76, 77). The GDF11-FTO-PPARγ axis prompted the shift of MSC lineage commitment to adipocyte and inhibited bone formation during osteoporosis, as a result of the imbalance between bone mass and fat. FTO expression resulted in the increase of the serum concentration of GDF11 in the bone, which was a key risk for osteoporosis. The GDF11-FTO signaling regulated the adipocyte and osteoblast differentiation of MSC by targeting PPARγ dependent of the m^6^A demethylase activity of FTO. Knock down the expression of FTO by means of lentivirus-mediated shRNA in BMSCs blocked the function of GDF11 and reduced the cells to differentiate to adipocytes. FTO knockout repressed the development of osteopenia *in vivo* through upregulation of adipocytic and down-regulation of an osteoblastic gene. FTO could regulate the m^6^A level of the transcriptional factor PPARγ mRNA. Aging and osteopenia were associated with a decline in m^6^A content in total RNA, which was consistent with the up-regulation of FTO expression (51). McMurray et al. conducted an evaluation to examine the effect of FTO demethylase function, and they found that pharmacologically inhibition FTO with IOX3 did significantly reduce BMD, BMC, and alter adipose tissue distribution. The level of alkaline phosphatase, an indicator of osteoblast function (61), was increased after use of IOX3 in mice compared with the controls (59). The process was revealed in Figure 2.

## Identification of m^6^A-associated SNPs for Bone Mineral Density or Osteoporosis

FTO polymorphisms are associated with elevated body mass index and increased risk for obesity (78, 79). Based on the candidate gene association study, FTO gene was found to be associated with hip fracture susceptibility (57). Specifically, researchers analyzed six single nucleotide polymorphisms (rs1421085, rs1558902, rs1121980, rs17817449, rs9939609, and rs9930506) of the FTO gene and found that female carriers of rs1121980 AA genotype had significantly higher risk of hip fracture with a hazard ratio of 2.06 (95% CI 1.17–3.62) than the female carriers of the wild-type. It was reported that ~17% of the variability in hip fracture risk was attributable to SNP rs1121980. The FTO gene might be a new candidate for BMD variation and osteoporosis in Chinese population, as a candidate genetic marker for peak bone mass acquisition (56). In Zhang's study, it was demonstrated that osteoblast expression of FTO was required for normal bone formation and maintenance of bone mass in mature mice. The results identified an epigenetic pathway in which FTO normally functioned in bone to enhance the stability of mRNA-encoding proteins that protected osteoblasts from genotoxic damage (80). Utilizing the Mendelian randomization analysis, it was founded that the FTO-BMI polymorphism (rs9939609), as an instrument, was significantly associated with total hip and femoral neck BMD but was not correlated with total spine BMD (81). In aggregate, it was revealed that FTO SNPs were not only associated with obesity and type 2 diabetes but also with the BMD at the hip (57, 79).

Currently, based on genome-wide association study, plenty of m^6^A-associated SNPs were identified as potential functional variants for BMD (52). Mo et al. found that 138, 125, and 993 m^6^A-SNPs were associated with the BMD of femoral neck, lumbar spine, and quantitative heel ultrasounds, respectively. Among them, the association between two genes (MIR196A2 and ESPL1) and BMD of lumbar spine reached the genome-wide significant level [rs11614913 (*P* = 8.92 × 10^−10^) and rs1110720 (*P* = 2.05 × 10^−10^), respectively] (52). Furthermore, expression quantitative trait locus analyses indicated that 47 of these BMD-associated m^6^A-SNPs were related with expressions of the 46 corresponding local genes. Besides, 24 m^6^A-SNPs were founded to be significantly associated with quantitative heel ultrasounds (*P* < 5.0 × 10^−8^) (52). This study provided new clues for further understanding of functional mechanism underlying the associations between SNPs and osteoporosis.

## M^6^A Modification in the Differentiation of Adipocyte and Osteoblast

Obesity and osteoporosis are closely correlated genetically (82–87). BMSCs is the same progenitor for adipocytes and osteoblasts and osteoblasts can also differentiate into adipocytes (87). Candidate genes, such as RANK (88), SP7 (89), and SOX6 (90), are all associated with obesity and osteoporosis. FTO affected not only obesity phenotypes, but also osteoporosis phenotypes, like BMD (57). FTO-knockout mice showed a significant reduction in adipose tissue and body lean mass (91), and in turn, reduced lean mass is associated with weaken femur bone strength (92). METTL3-mediated m^6^A RNA methylation also participated in the delicate process between pBMSCs adipogenesis differentiation and osteogenic differentiation (53–55, 66). BMI might be the causative role in osteoporosis the same as osteoarthritis on the effect of FTO variation (93). Recently, using a Mendelian randomization approach, Kemp et al. found that fat mass/BMI was strongly positively related to increased bone mineral density of the limbs, pelvis, and spine, but not the skull. In contrast, they reported that no evidence showed BMD could causally affect BMI or measures of adiposity (94). Taken together, m^6^A modification was closely related to the differentiation of adipocyte and osteoblast, which was important for the pathological development of osteoporosis.

## Concluding Remarks

Osteoporosis is a major public health concern with growing prevalence. Studies have indicated the important role of m^6^A modification in prevention, treatment and management of osteoporosis; however, more endeavors are needed to further understand the mechanism and clarify the relationship between m^6^A modification and osteoporosis.

## Author Contributions

All authors listed have made a substantial, direct and intellectual contribution to the work, and approved it for publication.

### Conflict of Interest

The authors declare that the research was conducted in the absence of any commercial or financial relationships that could be construed as a potential conflict of interest.
